# Identification of recurrent and novel mutations in *TULP1* in Pakistani families with early-onset retinitis pigmentosa

**Published:** 2012-05-10

**Authors:** Muhammad Ajmal, Muhammad Imran Khan, Shazia Micheal, Waqas Ahmed, Ashfa Shah, Hanka Venselaar, Habib Bokhari, Aisha Azam, Nadia Khalida Waheed, Rob W.J. Collin, Anneke I. den Hollander, Raheel Qamar, Frans P. M. Cremers

**Affiliations:** 1 Department of Biosciences, Faculty of Science, COMSATS Institute of Information Technology, Islamabad, Pakistan; 2 Department of Human Genetics, Radboud University Nijmegen Medical Centre, Nijmegen, The Netherlands; 3 Shifa College of Medicine, Islamabad, Pakistan; 4 Centre for Molecular and Biomolecular Informatics, Radboud University Nijmegen Medical Centre, Nijmegen, The Netherlands; 5 Nijmegen Centre for Molecular Life Sciences, Radboud University Nijmegen Medical Centre, Nijmegen, The Netherlands; 6 Institute of Ophthalmology, Mayo Hospital, Lahore, Pakistan; 7 Shifa International Hospital, Islamabad, Pakistan; 8 Department of Ophthalmology, Radboud University Nijmegen Medical Centre, Nijmegen, The Netherlands

## Abstract

**Purpose:**

To identify the genetic defects underlying retinitis pigmentosa (RP) in Pakistani families.

**Methods:**

Genome-wide high-density single-nucleotide-polymorphism microarray analysis was performed using the DNA of nine affected individuals from two large families with multiple consanguineous marriages. Data were analyzed to identify homozygous regions that are shared by affected sibs in each family. Sanger sequencing was performed for genes previously implicated in autosomal recessive RP and allied retinal dystrophies that resided in the identified homozygous regions. Probands from both families underwent fundus examination and electroretinogram measurements.

**Results:**

The tubby-like protein 1 gene (*TULP1*) was present in the largest homozygous region in both families. Sequence analysis identified a previously reported mutation (c.1138A>G; p.Thr380Ala) in one family and a novel pathogenic variant (c.1445G>A; p.Arg482Gln) in the other family. Both variants were found to be present in a homozygous state in all affected individuals, were heterozygous present in the unaffected parents, and heterozygous present or absent in normal individuals. Affected individuals of both families showed an early-onset form of RP.

**Conclusions:**

Homozygosity mapping, combined with candidate-gene analysis, successfully identified genetic defects in *TULP1* in two large Pakistani families with early-onset retinitis pigmentosa.

## Introduction

The major cause of inherited blindness in humans is retinitis pigmentosa (RP; OMIM 268000). The clinical symptoms of RP are the loss of night vision in the early phase of disease, later followed by peripheral vision loss, tunnel vision, and sometimes complete blindness [1]. Progression of the disease is mainly caused by the gradual loss of rod photoreceptor cells, which are mostly responsible for vision under low light conditions, and the subsequent loss of cone photoreceptor cells, which are involved in color vision under bright light conditions. The clinical diagnosis is based on fundus examination and electrophysiological analysis of rod and cone photoreceptor-cell function by measuring the scotopic and photopic responses, respectively, using electroretinography (ERG). The disease’s characteristics are the presence of pigmentary deposits (bone spicules) in the peripheral fundus, diminished or no ERG responses from rod and cone photoreceptor cells, and attenuation of the retinal blood vessels [1].

The disease is highly genetically heterogeneous, since 55 different genes and three loci have been identified as being associated with nonsyndromic RP (RetNet, Nov. 8, 2011). All Mendelian forms of inheritance have been observed for RP. Digenic forms and rare mitochondrial inheritance have also been reported [1-4].

Tubby-like protein 1 (TULP1; OMIM 602280) belongs to the tubby-like protein family. The *TULP1* gene is located on the short arm of chromosome 6 [5]. *TULP1* is expressed specifically in the retina [5,6], and the encoded protein is thought to be involved in protein trafficking, such as the transport of rhodopsin from the inner segment to the outer segment via the connecting cilium [7].

The aim of the current study was to identify the underlying genetic causes of autosomal recessive RP (arRP) in Pakistani families by using genome-wide homozygosity mapping and Sanger sequencing of known retinal disease genes in the homozygous regions. We identified disease-causing mutations in *TULP1* in two large families.

## Methods

### Ethics committee/institutional review board approval

Before initiating this study, approval for this work was granted by the Ethics Committee/Institutional Review Board of the Shifa College of Medicine/Shifa International Hospital, Islamabad, Pakistan, and signed informed consent was obtained from all participating individuals in both families.

### Ascertainment of families and clinical analysis

**Figure 1:**
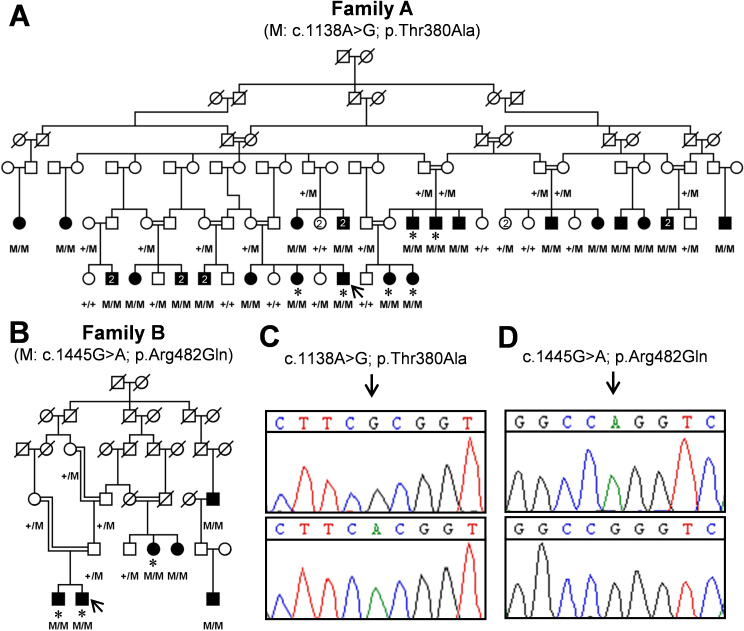
Pedigrees and *TULP1* genotyping results for families A and B. **A**: This is the pedigree of family A in which the presence of the c.1138A>G variant (M) was found in a homozygous state in all affected individuals. As expected for causal autosomal recessive variants, unaffected parents are heterozygous, and normal individuals carry one or no mutant allele. **B**: This is the pedigree of family B in which the presence of the c.1445G>A variant (M) was shown in a homozygous state in the 6 affected individuals, and heterozygously in an unaffected sibling, parents and grandparents of affected persons. **C**: This is the sequence electropherogram showing the nucleotide change from adenine to guanine in the proband of family A (upper panel), and sequence electropherogram of a control individual showing the wild-type adenine (lower panel). **D**: This is the sequence electropherogram of the proband carrying the mutant adenine in family B (upper panel); and the sequence electropherogram of a control individual with the normal guanine (lower panel). Probands are indicated with arrows; asterisks indicate the individuals that were tested using HumanOmniExpress (>700K) SNP microarrays. M/M, homozygous c.1138A>G (in family A) or homozygous c.1445G>A (in family B); +/M, heterozygous mutations present; +/+, two wild-type alleles present.

Two families from the Punjab province with individuals having night vision and daytime vision loss were included in the study. Their pedigrees were drawn (Figure 1A,B) using Haplopainter [8], and venous blood samples were collected in acid citrate dextrose-containing vacutainers (Becton Dickinson, Franklin Lakes, NJ). Fundus examinations were done, and ERG [9] measurements were made for probands in both families.

### Genotype analysis and homozygosity mapping

DNA was extracted by using a standard phenol chloroform extraction protocol [10] and stored at 4 °C. HumanOmniExpress (>700 K) single nucleotide polymorphism (SNP) microarrays from Illumina Inc. (San Diego, CA) were employed to search for homozygous regions in six affected individuals (Figure 1A) in family A and three affected individuals (Figure 1B) in family B. Genotype data were analyzed with Homozygosity Mapper [11], an online tool for homozygosity mapping using SNP genotyping data. Haplotype comparisons were also done for affected and normal individuals to identify the homozygous regions that were identical in all the affected individuals in each family.

### Sequence analysis and mutation detection

Prior to Sanger sequencing, the 15 protein-coding exons of *TULP1* and their flanking intronic sequences were amplified by PCR using standard conditions and reagents. PCR primers were designed with the online primer-designing tool Primer3 [12] (available on request). Amplified PCR products were electrophoresed in 2% agarose gels containing ethidium bromide, and DNA bands were visualized on an ultraviolet transilluminator. PCR products were purified on PCR clean-up purification plates (NucleoFast™ 96 PCR, Cat. No. 743100.10; MACHEREY-NAGEL, Düren, Germany), according to the manufacturer’s protocol. Purified PCR products were subsequently used for Sanger sequencing in an automated DNA sequencer (Big Dye Terminator, version 3, on a 3730 DNA analyzer; Applied Biosystems, Foster City, CA).

Sequencing results were analyzed by using Vector NTI Advance™ 2011 software from Life Technologies/Invitrogen (Bleiswijk, Netherlands), by assembling the sequenced contigs and then visualizing the aligned sequences of the exons.

### Pathogenicity assessment of identified variants

Identified missense variants were assessed for possible causality by using sorting intolerant from tolerant (SIFT) analysis and polymorphism phenotyping (Polyphen).

### Restriction fragment length polymorphism analysis

Restriction fragment length polymorphism analysis was performed to detect the presence or absence, of the identified mutations, in 100 ethnically matched control individuals. For the mutation identified in family A, restriction enzyme HpyCH4III was used, whereas in family B, restriction enzyme MspI was used. In both families, restriction enzyme recognition sites were abolished in the mutant sequences. Purified PCR products were used for restriction enzyme digestion, according to the manufacturer’s protocol (New England BioLabs, Ipswich, MA).

### Evolutionary conservation of amino acids

To check the evolutionary conservation of the mutated amino acids, the TULP1 orthologous protein sequences of the following species were aligned with the Vector NTI Advance™ 2011 software: humans (*H. sapiens,* ENSP00000229771); chimpanzees (*P. troglodytes,* ENSPTRP00000007764); mice (*M. musculus*, ENSMUSP00000049070); dogs (*C. familiaris,* ENSCAFP00000001922); chickens (*G. gallus,* ENSGALP00000009613); frogs (*X. tropicalis,* ENSXETP00000000899); tetraodons (*T. nigroviridis,* ENSTNIP00000004001); fruitflies (*D. melanogaster,* FBpp0088961); honeybees (*A. mellifera,* GB19892-PA); roundworms (*C. elegans,* F10B5.4), blood flukes (*S. mansoni,* Smp_058730__mRNA); and Arabidopsis (*A. thaliana,* AT1G76900.1).

### Three-dimensional structure prediction

Project HOPE [13] was used to predict the possible structural changes in the mutant TULP1 proteins identified in our study using a normal TULP1 structure (PDB-file 3C5N).

## Results

**Figure 2:**
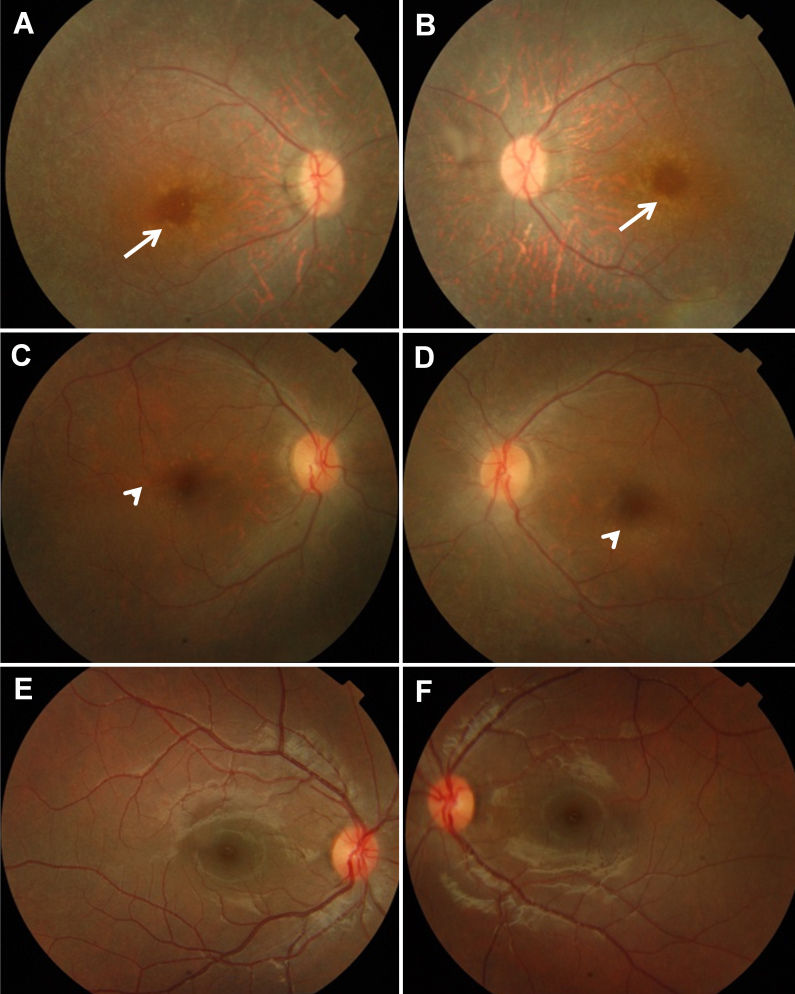
Fundus photographs of affected individuals from both families and of a normal individual. **A**, **B**: Right and left fundus, respectively, of the proband of family A (see arrow, Figure 1A), representative of the fundus appearance of all affected members of this family. Arrows indicate yellow perifoveal annular rings. **C**, **D**: Right and left fundus, respectively, of the proband of family B (see arrow, Figure 1B). Arrowheads point to the developing perifoveal annular rings. **E**, **F**: Right and left fundus, respectively, of a normal individual.

In both families, the average age of disease onset was in the first decade of life. Ophthalmic examination of affected individuals from both families revealed the presence of attenuated retinal vessels and the optic disc to have a waxy, pale appearance (Figure 2). A yellow perifoveal annular ring, a characteristic feature of individuals carrying *TULP1* mutations, was also clearly visible in family A (Figure 2A,B). In family B, the perifoveal ring was in the process of development (Figure 2C,D). Upon ERG, scotopic and photopic electrophysiological responses of rod and cone photoreceptors, respectively, were diminished in affected members of both families (Table 1). Neither nystagmus nor eye poking were present in either family.

**Table 1:** Comparison of ERG responses of affected individuals of families A and B with a control individual.

**Measured parameters using monopolar electrodes**	**Adaptation**	**Flash strength (cds/m2)**	**Proband Family A**	**Proband Family B**	**Control**	**Normal values (Age=20 years)**
Scotopic 25 dB b-wave amplitude (µV)	Dark	0.01	13.3	21.8	244.4	>185
Scotopic 0 dB b-wave amplitude (µV)	Dark	3	22.7	22.2	650	>419
Oscillatory potential amplitude (µV)	Dark	3	56.4	47.9	187.7	>110
Photopic 0 dB b-wave amplitude (µV)	Light	3	12	15.7	86.9	>102
Photopic 30 Hz flicker amplitude (µV)	Light	3	6.1	1.46	65.6	>70

Age of affected individuals from families A and B at the time of investigation was 20 years.

**Figure 3:**
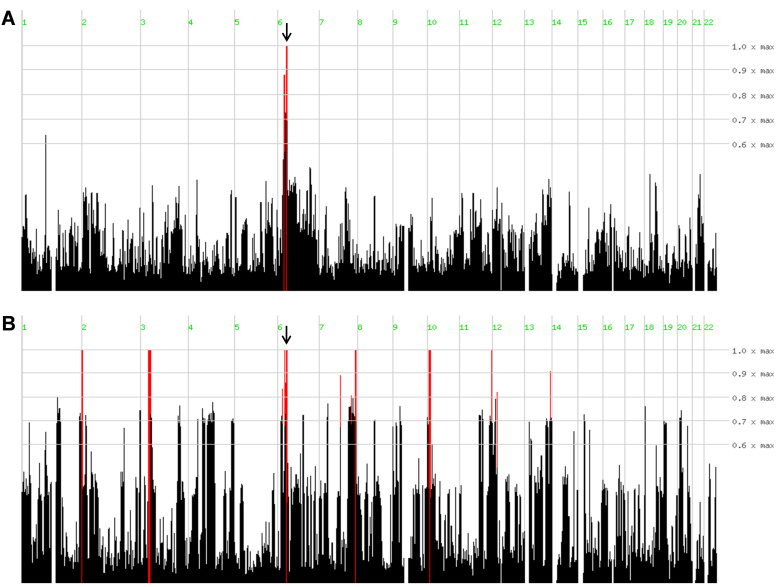
Homozygosity mapping results for families A and B. Homozygosity-mapper plots show the homozygous regions in the affected individuals in both families (red lines). Homozygous regions that show identical haplotypes for all affected individuals within a family are indicated by arrows. Panel **A** represents family A and panel **B** represents family B.

Genome-wide SNP microarray data of six affected individuals of family A were analyzed with the help of homozygosity mapper, which revealed a single homozygous region (Figure 3A) of 3.9 Mb (from 32.9 Mb to 36.8 Mb; hg19) on chromosome 6, flanked by SNPs rs3132131 and rs236411. This homozygous region harbored *TULP1*, a gene known to be mutated in patients with Leber congenital amaurosis (LCA) and arRP. The sequence analysis identified a previously reported mutation, c.1138A>G (p.Thr380Ala) [14,15] in this family (Figure 1C).

Similarly, genome-wide SNP microarray data analysis of three affected individuals of family B, resulted in the identification of six homozygous regions (Figure 3B). After haplotype comparison, two regions, a 4.8 Mb region on chromosome 6 (from 33.8 Mb to 38.6 Mb, flanked by SNPs rs9296102 and rs7761629) and a 1.4 Mb region on chromosome 7 (from 132.3 Mb to 133.7 Mb, flanked by SNPs rs924368 and rs10249912), were found to be identical in all the affected individuals. *TULP1* resides in the homozygous region on chromosome 6, and the sequence analysis revealed a novel mutation, c.1445G>A (p.Arg482Gln; Figure 1C). The homozygous chromosomal region on chromosome 7 did not contain a gene previously implicated in an inherited retinal disease such as arRP or LCA.

**Figure 4:**
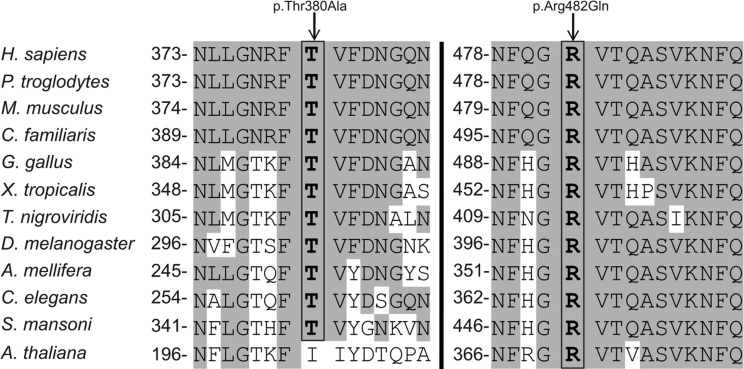
Conservation of mutated amino acids among different species. Amino acids identical to human tubby-like protein 1 (TULP1) amino acids are shaded in gray. The positions of the amino acids in the respective polypeptides are indicated.

In families A and B, the variants c.1138A>G and c.1445G>A, respectively, were found to be present in a homozygous state in all affected individual, were heterozygous present in the unaffected parents, and absent or heterozygous present in normal individuals (Figure 1A,B). Both wild-type nucleotides were shown to be highly conserved, as evidenced by their phylogenetic p value [16] scores of 2.87 (c.1138A) and 6.10 (c.1445G) for family A and B, respectively. In addition, the encoded amino acids, p.Thr380 and p.Arg482, located in the C-terminal tubby domain, are highly conserved among different vertebrate and invertebrate species, while in a plant (Arabidopsis), isoleucine is present instead of threonine (Figure 4). These amino acids are completely conserved among the tubby, TULP1, TULP2, and TULP3 proteins [17]. SIFT predicts that both TULP1 variants are “not tolerated” whereas Polyphen predicts that both are “probably damaging” with prediction scores of 0.827 and 1.000 for p.Thr380Ala and p.Arg482Gln, respectively.

**Figure 5:**
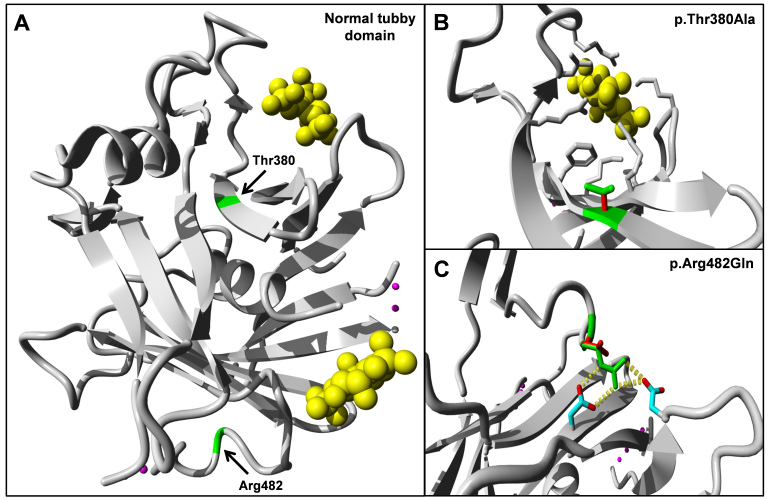
Three-dimensional domain architecture of the tubby domain of TULP1 wild-type and mutant proteins. **A**: Preferred predicted secondary structure of normal tubby-like protein 1 (TULP1) with Thr380 and Arg482 indicated in green. In yellow, the inositol triphosphate molecules that are predicted to bind the tubby domain of TULP1. **B**: Predicted structure of part of the p.Thr380Ala mutant protein in affected individuals of family A. The smaller size of the alanine residue may lead to rearrangements of surrounding residues and thereby affect putative inositol triphosphate binding. **C**: Part of the predicted structure of the p.Arg482Gln mutant protein found in affected individuals of family B. The p.Arg482Gln variant changes a positively charged amino acid (arginine) to a neutral residue (glutamine), which leads to loss of interactions with two negatively charged residues in its vicinity. Wild-type interactions are indicated with yellow blocks.

A three-dimensional-structure prediction analysis by project HOPE predicts that the p.Thr380Ala mutation, due to the smaller size of the alanine residue, causes an empty space in the protein and possible rearrangements of surrounding residues (Figure 5A,B). Any hydrogen bonds made by threonine will also be lost, because alanine is a hydrophobic residue. Very close to Thr380 is a predicted inositol triphosphate binding site that might also be influenced by local conformational changes. The p.Arg482Gln variant changes a positively charged amino acid (arginine) to a neutral residue (glutamine), which results in the loss of interactions with negatively charged residues in its vicinity (Figure 5C). In view of its location in the three-dimensional structure, these changes may result in a loss of external interactions.

The p.Thr380Ala and p.Arg482Gln variants were not detected in 100 healthy ethnically matched control individuals.

## Discussion

In the current study, we report on two large families with several consanguineous marriages and multiple individuals with early-onset RP that were found to carry *TULP1* mutations. Family A had 27 affected individuals who resided in the northern part of the Punjab province. This family had a strong custom of marrying inside the family. *TULP1* mutations were previously reported to cause LCA, a congenital form of severe vision impairment or blindness and early-onset RP [14,15,18-31]. There is clinical and genetic overlap between (early-onset) arRP and LCA [32,33], and often it is very difficult to differentiate between these two conditions. Mutations in Crumbs homolog 1 (*CRB1*), Lecithin retinol acyltransferase (*LRAT*), Mer tyrosine kinase protooncogene (*MERTK*), Retinal pigment epithelium-specific protein, 65-KD (*RPE65*), Spermatogenesis-associated protein 7 (*SPATA7*), and *TULP1* have been identified in individuals with LCA and arRP [18-21,34-47]. If a severe form of rod–cone dystrophy is observed beyond early childhood, and when there are no features reminiscent of LCA, such as nystagmus, or eye poking, the phenotype is classified as early-onset RP.

TULP family proteins (TUB, TULP1, TULP2, and TULP3) have crucial roles in embryonic development in vertebrates and take part in the proper functioning of the central nervous system [6,48]. TULP1 is expressed specifically in the rod and cone photoreceptor cells [5,6], and is involved in the transport of rhodopsin [7]. In *Tulp1*^−/−^ mice, photoreceptor degeneration precedes synaptic malfunction, and thus TULP1 may have a function in photoreceptor synapse development [49]. A mutation of the same residue (p.Arg482Trp) was earlier found in combination with p.Leu504fsX140 in five affected individuals with severe early-onset RP [50].

The structural analyses of the TULP1 C-terminal domains of the mutant proteins suggest that the missense mutations identified in our study might have resulted in the destabilization of the mutant proteins, or might have influenced the putative interactions of the tubby domain. Among different species of animals and plants, p.Arg482 is located in the signature sequence (F-[KRHQ]-G-R-V-[ST]-x-A-S-V-K-N-F-Q) of the Tubby family of proteins, and this signature sequence contains 11 invariant amino acids that are highly conserved (Prosite) [5]. Replacement of the wild-type residue with the mutant glutamine might affect the signature sequence; this might ultimately prevent the mutant TULP1 protein from functioning normally.

In family A, the presence of a typical yellow-colored perifoveal annular ring was also indicative of *TULP1* involvement [24]; whereas in family B, the ring formation was incomplete (Figure 2C,D). The bone spicules absent from both families might still develop later in life. The age of both individuals who were clinically evaluated was 20 years.

The previously identified mutation p.Thr380Ala has only been reported in two unrelated Pakistani families [14,15]. Our findings concerning this mutation in yet another Pakistani family suggested that c.1138A>G might be a Pakistani founder mutation, although no link was established between any of these families. One family belonged to a northern area of Pakistan [14] while the other belonged to the southern part of Punjab [15]. Our family belonged to the northern part of Punjab, which, however, does not exclude the possibility that this mutation represents a founder mutation in Pakistan.

**Figure 6:**
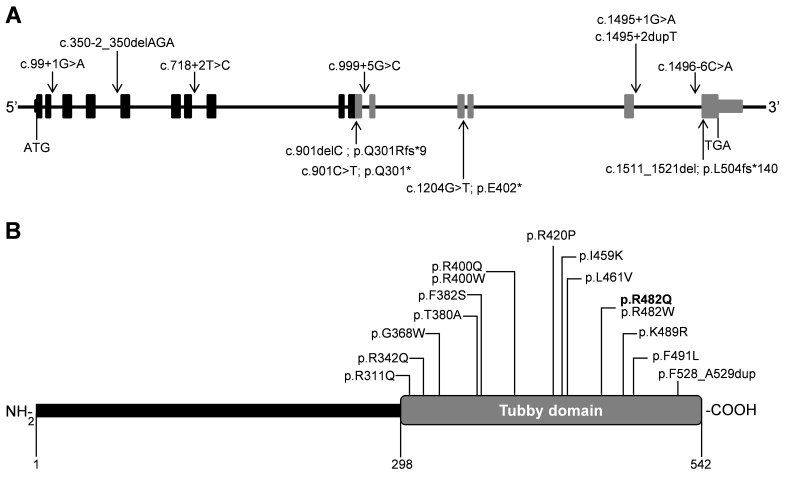
*TULP1* gene, protein structure, and pathologic variants identified in patients with LCA and arRP. **A**: Gene organization and distribution of splice site variants (above the gene) and nonsense and frame-shift variants (below the gene). **B**: Protein domain structure of tubby-like protein 1 (TULP1) showing missense changes and a two-amino-acid duplication in the tubby, C-terminal domain. The novel missense variant is indicated in bold.

Including our findings, 26 different *TULP1* mutations have been identified in 33 families (Table 2). *TULP1* mutations were found in 4.3% (10/231) of LCA families [14,18,24,25,27,29,30] and 2.4% (23/948) of arRP families [15,19-24,26,28,30,31,50]. *TULP1* mutations causing arRP or LCA include two nonsense, two frame-shift, and seven splice-site mutations; a six-base-pair duplication; and 14 missense mutations (Table 2). Splice-site and protein-truncating mutations are distributed throughout the gene (Figure 6A), whereas the missense mutations are only present in the C-terminal tubby domain of TULP1 (Figure 6B).

**Table 2:** *TULP1* mutations causing arRP or LCA.

**Exon/intron**	**Mutations: Allele 1/Allele 2**	**Phenotype**	**Number of families**	**Number of cases**	**Reference**
Intron 2, Exon 12	c.99+1G>A/c.1204G>T(p.Glu402*)	LCA	1/179	1/179	[18]
Intron 4, Exon 5, 10	c.350–2_350delAGA/c.901delC (p.Gln301Argfs*9)	arRP	1/49	2/49	[22]**
Intron 2, Exon 14	c.99+1G>A/c.1376T>A (p.Ile459Lys)	arRP	1/536	1/536	[20]
Intron 7	c.718+2T>C/c.718+2T>C	juvenile RP	2/86	286	[24]
Exon 10	c.901C>T (p.Gln301*)/c.901C>T (p.Gln301*)	LCA	5/37	42/117	[29]
Exons 10, 11	c.932G>A (p.Arg311Gln)/c.1025G>A (p.Arg342Gln)	arRP	1/2	2/4	[31]
Intron 10	c.999+5G>C/c.999+5G>C	juvenile RP	1/86	1/86	[24]
Exon 11	c.1102G>T (p.Gly368Trp)/c.1102G>T (p.Gly368Trp)	LCA	1/179	1/179	[18]
Exon 12	**c.1138A>G (p.Thr380Ala)/c.1138A>G (p.Thr380Ala)**	LCA, early onset arRP	1/14, 1/5, 1/2	3/64, 4/23, 27/33	[14,15] and this study
Exon 12	c.1145T>C (p.Phe382Ser)/c.1145T>C (p.Phe382Ser)	arRP	1/59	2/59	[23]
Exon 12	c.1199G>A (p.Arg400Gln)/c.1199G>A (p.Arg400Gln)	arRP	1/34	2/34	[28]
Exon 12	c.1198C>T (p.Arg400Trp)/c.1198C>T (p.Arg400Trp)	LCA	1/179	1/179	[18]
Exons 13, 14	c.1259G>C (p.Arg420Pro)/c.1471T>C (p.Phe491Leu)	arRP	1/536	2/536	[20]
Exon 14	c.1381C>G (p.Leu461Val)/c.1381C>G (p.Leu461Val)	juvenile RP	1/86	1/86	[24]
Exon 14	**c.1445G>A (p.Arg482Gln)/c.1445G>A (p.Arg482Gln)**	early onset arRP	1/2	6/33	This study
Exon 14	c.1466A>G (p.Lys489Arg)/c.1466A>G (p.Lys489Arg)	early onset arRP, arRP	4/5, 1/171	19/23, 1/171	[15,19]***
Intron 14	c.1495+1G>A/c.1495+1G>A	arRP	2/2	33/33	[21]
Intron 14	c.1495+2dupT/c.1495+2dupT	early onset arRP	1/1	3/3	[26]
Intron 14	c.1496–6C>A/c.1496–6C>A	arRP	1/171	1/171	[19]
Exons 14, 15	c.1444C>T (p.Arp482Trp)/ c.1511_1521delTGCAGTTCGGC (p.Leu504fs*140)	early onset arRP	1/1	5/5	[50]
Exon 15	c.1582_1587dupTTCGCC (p.Phe528_Ala529dup)/ c.1582_1587dupTTCGCC (p.Phe528_Ala529dup)	LCA	1/1	7/7	[25]****

**Variant labeled as c.937delC. ***Same change at protein level but labeled as c.1502G>A at the cDNA level. ****Change labeled as c.1593_1594TTCGCC (FA531–532dup). The variants identified in this study are marked in bold. Patients with visual loss, nystagmus and night blindness after the age of six months but not later than six years are diagnosed as juvenile RP [24].

*TULP1* mutations are a frequent cause of LCA and arRP, and therefore represent an attractive therapeutic target. Thus far, *TULP1* mutations have been found in a total of 136 individuals with LCA or arRP (Table 2). Through our studies, 33 additional patients with *TULP1* mutations might benefit from genetic counseling and future gene-augmentation therapy.

In conclusion, we were able to identify one novel (c.1445G>A; p.Arg482Gln) and one previously identified (c.1138A>G; p.Thr380Ala) disease-causing mutation in *TULP1* in Pakistani families with early-onset RP.
